# Role of Aquaporins in a Composite Model of Water Transport in the Leaf

**DOI:** 10.3390/ijms17071045

**Published:** 2016-06-30

**Authors:** Adi Yaaran, Menachem Moshelion

**Affiliations:** Faculty of Agriculture, Food and Environment, The Robert H. Smith Institute of Plant Sciences and Genetics in Agriculture, The Hebrew University of Jerusalem, Rehovot 76100, Israel; adiyaaran@gmail.com

**Keywords:** hydraulic conductance, turgor, transcellular water movement, membrane osmotic permeability (P_f_)

## Abstract

Water-transport pathways through the leaf are complex and include several checkpoints. Some of these checkpoints exhibit dynamic behavior that may be regulated by aquaporins (AQPs). To date, neither the relative weight of the different water pathways nor their molecular mechanisms are well understood. Here, we have collected evidence to support a putative composite model of water pathways in the leaf and the distribution of water across those pathways. We describe how water moves along a single transcellular path through the parenchyma and continues toward the mesophyll and stomata along transcellular, symplastic and apoplastic paths. We present evidence that points to a role for AQPs in regulating the relative weight of each path in the overall leaf water-transport system and the movement of water between these paths as a result of the integration of multiple signals, including transpiration demand, water potential and turgor. We also present a new theory, the hydraulic fuse theory, to explain effects of the leaf turgor-loss-point on water paths alternation and the subsequent reduction in leaf hydraulic conductivity. An improved understating of leaf water-balance management may lead to the development of crops that use water more efficiently, and responds better to environmental changes.

## 1. A Composite Model of Water Transport in the Leaf

Land plants evolved 450 million years ago from aquatic algae [1]. Vascular plants have evolved adaptations to extreme differences in environmental conditions while competing for light. Land plants’ dependence on soil water increases the need for efficient water transport along the soil-plant-atmosphere continuum (SPAC), making efficient hydraulic conductance regulation and dynamic response to the environment extremely advantageous [2,3,4,5,6,7]. Stomatal aperture governs the exchange of gases between the mesophyll cells (MCs) and the atmosphere. Wide apertures enable CO_2_ uptake, as well as the simultaneous loss of water. Therefore, sufficient hydraulic conductance of the vascular tissue can allow for greater water loss via open stomata, as well as increased CO_2_ assimilation [2,5,8,9]. In fact, higher crop yields have been correlated with increased stomatal conductance [10], high leaf hydraulic conductivity (K_leaf_) [5] and greater water loss. K_leaf_ changes in response to environmental factors such as stress and light [5,11,12,13,14,15,16,17,18]. Water moves through the leaf across a number of tissues via several parallel paths. Despite the great efforts that have been made to clarify our understanding of the regulation of water movement in the leaf and the relative weight of each path, that understanding remains elusive.

The movement of water through the plant has been compared with the movement of current through an electrical circuit [5,19,20,21,22,23]. In that analogy, the potential difference between soil and atmosphere are analogous to the driving electrical potential in a circuit and water flux is analogous to electron flux based on a steady-state assumption. However, this analogy may be too simplistic, as the rates at which water enters and exits a plant are not entirely equal, which causes fluctuations in the plant’s water status (relative water content and water potential) over the course of a typical day, as well as in response to stress [24,25,26,27,28].

An alternative hydraulic flow model was recently suggested [29]. In this model, the leaf hydraulic resistance is not constant, but dynamic, and varies nonlinearly with water potential. This dynamic capability is controlled by aquaporins (AQPs) that are part of the plasma membrane (PIPs). In this hydraulic model, AQPs play a crucial role in the regulation of hydraulic conductance, aided by their quick reaction time (seconds to minutes), and the cell vacuole is the main water capacitor buffering against changes in the volume of the cytoplasm. It is important to mention that in addition to water, AQP were shown to facilitate the diffusion of additional small neutral solutes, including CO_2_. Therefore AQP presence and activity may effect CO_2_ transport and carbon metabolism (reviewed by [14,30,31,32,33]). Nevertheless, this review will focus on water movement in the leaf.

In 1997, Ernst Steudle suggested a composite transport model for the movement of water through the root [34]. That model suggests that the movement of water between parallel radial pathways (i.e., apoplast, symplast and transcellular) can be regulated by switching the apoplast path on and off. Moreover, the high degree of variability in the hydraulic resistance of a given root can be explained by the dynamics of the forces involved in moving water. In other words, the model presents dynamic regulation of water uptake in response to shoot demand (transpiration). In this review, we will try to use Steudle’s composite transport model to describe the movement of water through the leaf, emphasizing the different compartments and factors involved in the regulation of leaf hydraulic conductance and the possible role of AQPs in the dynamic regulation of the movement of water through the leaf.

## 2. Hydraulic Regulation of the Xylem and Leaf Veins

Tracheids and vessel elements are highly modified cells that have no membranes and are arranged to provide low-resistance axial pathways for apoplastic water transport. Venation architecture varies widely among plants and tends to be phylogenetically conserved [6,9,35,36]. The contribution of vascular components to the movement of water through the leaf (i.e., minor and major veins) also varies within species and can influence leaf and whole-plant hydraulic conductance [5,9,36,37]. For example, vein density can limit transpiration [6], providing an hydraulic link between venation and the rate of photosynthesis [2]. Hierarchical reticulate and redundant venation patterns are common among angiosperms, providing vascular-mesophyll economic constraints and risk tolerance [35,36]. Xylem structure can be altered by environmental factors such as water stress [38,39,40], irradiance [9,40,41,42] and CO_2_ concentration [43]. However, such adjustments occur relatively slowly since they require developmental changes. This passive structural conductance pathway might be a bit more dynamic due to the pit membrane. The biochemical composition, macromolecular structure and hydraulic conductance of this membrane may change in response to ion concentrations in the xylem sap, to control the hydraulic properties of the vessel [44,45]. The vascular architecture and structure may play a less dominant role with regard to the rapid and dynamic regulation of K_leaf_.

In this review, we present studies that point to a cellular regulation mechanism that plays a central role in controlling leaf vascular radial conductance. This rapid regulation is most likely controlled by the membrane selectivity of the parenchymatic cells that surround the dead trachea elements and control the radial flow of water from the vessels into the leaf MCs.

## 3. The Leaf Vascular Bundle Sheath Cells (BSC) as a Selective Barrier

Leaf bundle sheath cells (BSCs) form a layer of compact parenchyma that surrounds the entire vasculature [46,47], except for the ends of the vessel elements, the hydathodes [47], and maintain hydraulic integrity along the vasculature [48]. In recent years, evidence has appeared to indicate that the bundle sheath acts as a dynamic hydraulic barrier around the xylem tracheids. The evidence is as follows:

1. No symplastic continuum appears to exist between the bundle sheath and the phloem and xylem [49].
2. Hydraulic pressure builds up in the xylem, usually at night, and is released only from the hydathodes (as guttation drops) and not through the vascular tissue (i.e., the air spaces within the mesophyll are flooded) [50].
3. Several plant bundle sheath cell walls include lipophilic components similar to the endodermal Casparian strips found in the root [5,51,52].
4. The fact that the bundle sheath is selectively permeable to small molecules such as Boron (B), Na^+^ and H_2_O_2_ [50,53,54,55] suggests that it may act as a xylem–mesophyll apoplastic barrier.

While the vascular system transports water axially throughout the leaf, the bundle sheath serves as a radial membrane gateway and may regulate the identity and quantity of substances transported between the vascular system and the rest of the leaf [5]. Thus, hydraulic isolation of the bundle sheath together with dynamic transport control could theoretically increase or decrease the efficiency of water transport.

The dynamic regulation of K_leaf_ by the AQPs of BSCs has been addressed in a few recent studies. Gene expression patterns of PIP2s showed dominant (PIP2;1) or exclusive (PIP2;6) expression in Arabidopsis veins [15]. In addition, immunohistochemical work revealed vacuolar and plasma membranes (PM) of *Brassica napus* BSCs contain more γ-TIP/VM 23 and PIP1 (respectively) than MCs do, and high PIP1 levels were observed in invaginations of vascular parenchyma plasmalemma (plasmalemmasome). These findings led those researchers to speculate that BSCs play an important role in facilitating the movement of water between the apoplastic and symplastic routes next to vascular tissues [56]. K_leaf_ generally increases as light intensity increases [9,12,13,57,58]. Indeed, light increases AQP transcript levels in walnut (*Juglans regia*) (blue light in particular in this study [13]) and also increases the hydraulic conductivity (L_p_) of *Zea mays* midrib parenchyma cells [59]. In addition, light-dependent phosphorylation of PIP2;1 in Arabidopsis BSCs has been linked to increased rosette conductivity (K_ros_) [15]. These findings further emphasize the hydraulic properties of the bundle sheath and K_leaf_ dynamic, suggesting a molecular mechanism (AQP) for the dynamic regulation of K_leaf_.

However, a different study found that light reduced the osmotic water permeability (P_f_) of BSCs while increasing K_leaf_ [60]. The authors of that study suggested that this may indicate a decrease in the hydraulic resistance of the leaf apoplast, but also indicated that more studies will be required to explain this response.

ABA was reported to affect BSC hydraulic properties, decreasing the P_f_ of BSCs by downregulating the activity of their AQPs [18]. The application of ABA through petioles decreased K_leaf_ and reduced transpiration [18,61]. In addition, reactive oxygen signaling processes integrating light and ABA signaling have been shown to be regulated within BSCs [55], yet the authors of that work did not refer to any role for AQP in that process.

It has been suggested that BSCs may also act as a control center to coordinate xylem hydraulic conductance with the hydraulic demand of MCs [5,62], strengthening the involvement of hydraulic signals in the regulation of leaf water balance [63]. The substantial effect of the BSC on the hydraulics of MCs was demonstrated by Sade [64], who reported that Bundle Sheath (BS)-specific silencing of several members of AQP family reduced the P_f_ of MCs, without reducing their conductance of CO_2_, suggesting that the BS-MC hydraulic continuum acts as a feed-forward control signal. Recently, it has also been suggested that reductions in water permeability within leaf vascular tissues indirectly induce stomatal closure via ABA signals or via a hydraulic (hydro-passive) signal [18,61,65,66].

The architecture, location, and biochemical and physiological properties of the BS enable radial water transport, apparently involving AQP, strongly supporting the BS’s role as a key regulatory hydraulic checkpoint that determines the rate at which water and minerals flow through the leaf. This radial hydraulic control plays a major role in controlling whole-leaf water balance.

**Figure 1:**
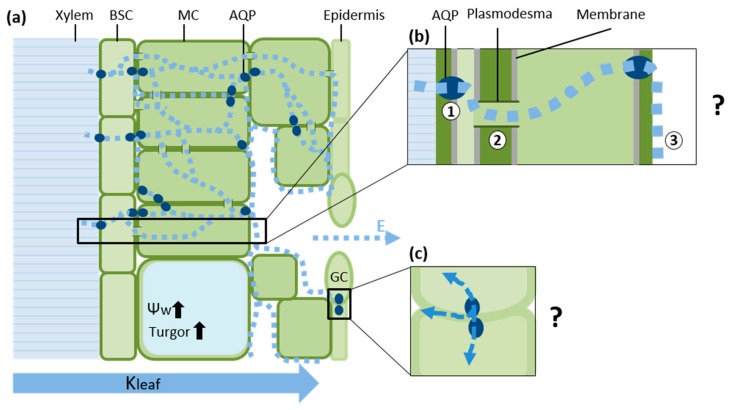
Composite model of water transport in the leaf. (**a**) From the vascular system, water (dashed line) is transported transcellularly via regulated Aquaporins (AQPs) into Bundle Sheath Cell (BSC). The amount of water allowed to enter the leaf is determined by hydraulic and chemical signals. If the amount of water moving out of the leaf (transpiration, E) is greater than the amount entering the leaf (via the BSC), a hydraulic signal can be induced or strengthened; (**b**) From the bundle sheath, water moves toward the mesophyll Cells (MCs) and Guard Cells (GCs) via three pathways: (1) a transcellular pathway; (2) the symplast (plasmodesmata) and (3) the apoplast. We propose that the relative amounts of water moving through each of these pathways can be altered by AQPs in response to changes in leaf water status, i.e., under optimal conditions (high water potential, Ψ_W_, and turgor), high levels of transpiration (E, dash line arrow) will encourage the transport of water through the apoplast by reducing the activity of AQPs in the mesophyll. In contrast, under less favorable conditions (plasmolysis, low Ψ_W_, and turgor), there may be an increase in AQP activity that encourages the transport of water through the symplast. However, the regulation of the distribution of water among these pathways is not yet understood; (**c**) Stomatal AQPs can affect the rate at which stomata open and close, in accordance with the turgor of neighboring cells. The destination of the liquid water that leaves the guard cells is unknown. This water may enter neighboring cells or the apoplast of the stomatal cavity, or be lost through transpiration. Hydraulic conductance of the leaf (K_leaf_).

However, the pathways between the BSC and stomata are not clear. Passing through the BS, water can proceed toward evaporation sites in the mesophyll via three alternative pathways: (1) a transcellular path via AQPs; (2) a symplastic cell-to-cell path via plasmodesmata and (3) an apoplastic path along the cell wall (Figure 1). The relative distribution of the quantities of water transported via each of these pathways is poorly understood and seems to vary by species, leaf structure, developmental stage and physiological conditions [5,14,33]

## 4. Hydraulic Properties of Mesophyll Cells

The leaf mesophyll is a spongy tissue that includes the majority of the leaf cells. The spongy structure of the mesophyll cells results in large surface areas facing air spaces inside the leaf, which facilitates CO_2_ uptake, as well as the loss of water. This extensive sub-epidermal surface area is up to 40 times greater than the leaf’s exterior surface area [67,68]. These extensive airspaces also limit contact between MCs, complicating apoplastic liquid water movement. However, it is important to note that MCs might not be homogenous in its hydraulic properties. Canny et al. [68] showed that the MCs of cotton (*Gossypium hirsutum*) includes shrinking cells (spongy and cavity cells) from which water evaporates, as well as non-shrinking cells (matrix cells) from which water probably does not evaporate, and suggested that evaporation is limited to only a portion of MCs.

Among terrestrial plants, the hydraulic conductivity of the MCs is consistently low. Therefore, we can presume that the length of the hydraulic pathway through the MCs influences K_leaf_ [2]. Plants have adapted different strategies for overcoming the hydraulic resistance of the mesophyll, such as a reticulated vein system to allow maximal proximity to evaporation sites, as well as more unique strategies such as a heterobaric leaves that include bundle sheath extensions that connect the epidermis and vascular bundles [69,70] or directed lignification and apoptosis of a proportion of the MCs [2]. All of these strategies provide ways to bypass the slow movement of water through live MCs, affecting K_leaf_. Yet, the exact contributions of these adjustments to the plant’s hydraulic balance are not fully understood.

Cell-to-cell water transport constitutes a significant part of the leaf hydraulic path from xylem to epidermis and AQPs may play a role in this pathway [71]. Numerous AQPs have been localized to the PM of MCs [33,72], including invaginations of the PM (plasmalemmasomes) [73].

The P_f_ of MCs varies widely, within the range of 0 to more than 60 µm·s^−1^, with the majority of cells exhibiting P_f_ levels between 0 and 10 µm·s^−1^ under control conditions [18,72,74]. A study that examined water loss from MCs of epidermis stripped leaf found that the hydraulic conductance of MCs varies approximately 5-fold over an approx. 24-h cycle, and the author of that study suggested that AQPs mediate the regulation of MCs to produce cyclic changes in the rates of water loss and transpiration [68]. Indeed, Arabidopsis MCs P_f_ was reduced in AtPIP1;2-knockout protoplasts [72] and was also dramatically reduced when the whole PIP1 subfamily was silenced [66], pointing to a possible role for AtPIP1;2 in MCs’ water transport.

Morillon et al. [74] showed that transpiration intensity affects the P_f_ of mesophyll cells, apparently due to the inactivation of AQPs, for example, an increase in transpiration suppresses the activity of AQPs in MCs, which lowers the P_f_ of those cells. This reduction in the use of the transcellular path is congruent with the suggested predominance of apoplastic water movement during transpiration [5,15,23,75,76], as well as an earlier suggestion that AQPs may be involved in the movement of water between the apoplast and symplast [77] or vacuole [73].

**Figure 2:**
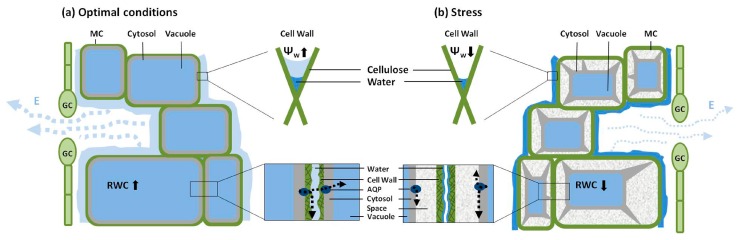
Mesophyll Cell (MC) turgor as a hydraulic fuse altering water path through the leaf. (**a**) When turgid, cells Plasmamembrane (PM) is in contact with cell wall so that water exiting trough AQP substantially wet cell wall, sustaining apoplastic water flow; (**b**) When water statues is low, plasmolysis cause distancing of PM from cell wall, and as a fuse, cuts off water supply to the cell wall. The negative water potential (Ψ_W_, arrows indicate high or low) generated within the microcapillary structure of the mesophyll cell walls serves as a transpiration (E) pump pulling water from the xylem [80]. This negative water potential is highest (absolute value) next to the mesophyll membrane and may serve as a pump (i.e., difference in water potentials between the cell wall and the protoplast) resulting in water leaving the cell until physical disconnection between the protoplast and its wall, at which point no further reduction in Relative Water Content (RWC, arrows indicate high or low) is observed. The water which evaporates from the cell wall leaves the leaf via the Guard Cells (GC).

In this way, AQPs may act as dynamic valves to modulate the movement of water between the three possible pathways. Accordingly, the AQP valves may control the wetting rate of the walls of MCs (assuming the PM of a turgid cell is in close contact with the cell wall). Hence, under stress conditions, when these cells reach their turgor loss point (TLP) and undergo plasmolysis, the separation of the PM from the cell wall limits the ability of AQP to sustain the apoplastic pathway, thereby acting as a *hydraulic fuse* (Figure 2). When the size of the area of contact between the PM and cell wall is reduced (yet the PM and endoplasmic reticulum remain in close contact with the plasmodesmatal pore, maintaining continuity between cells via the central desmotubules [78,79]), cell-wall wetting will be reduced and this will affect the apoplastic pathway, regardless of any AQP activity. Transpiration serves as a pump, generating a negative water potential within the cell wall microcapillary of MCs, pulling water from the xylem. Water will continue to leave the cell until the protoplast physically disconnects from the cell wall [80]. Furthermore, as Morillon et al. [74] suggested, AQP can play a role in the movement of water between neighboring cells. According to this theory, the TLP acts as a hydraulic fuse, distancing AQP from the cell wall. This provides over-flux protection to MCs and sharply reduces the cell wall water potential (as described by the capillarity model [81]), which might serve as a signal for stomatal closure.

Interestingly, a global meta-analysis revealed that relative water content (RWC) at the TLP is a strongly preserved parameter across many plant species [82]. TLP was previously proposed to act as a notable hydraulic stress signal in plant water balance, possibly triggering an ABA signal in the shoot [63,83]. Moreover, a very recent study showed that biosynthesis of ABA is triggered by a reduction in leaf turgor in angiosperms, and turgor pressures that trigger increases in foliar ABA correlate with Ψ_tlp_. However, in that study, foliar ABA levels significantly increased when leaf turgor was positive, before the turgor loss point was reached [84]. Control of the rate of cell-wall wetting by PM AQPs in accordance with plasmolysis rate may be an additional outcome of the cell TLP-signaling mechanism, acting together or in parallel with mechanical sensing of water balance. However, it is not clear whether the gaps formed between PM and the cell wall during plasmolysis are filled with liquid or gases, so this hypothesis should be considered with skepticism.

## 5. Permeability of Guard Cells to Water and Regulation of Stomatal Aperture

Water leaves the plant through the stomatal pore as vapor. The site of evaporation and the role of AQPs in this process are still not clear and may vary according to leaf anatomy (reviewed by [3,5]). Nevertheless, stomatal movement requires the transport of liquid water from and into guard cells, aside from the movement of water vapor. Guard cells are an integral part of the epidermis and changes in their volume are partially controlled by subsidiary cells that play a mechanical role as their turgor pressure restricts the distancing of guard cells from one another, thereby affecting stomatal apertures [85,86].

It is important to mention the relationship between transpiration and plant hydraulic conductance [2,5,24,27,38,61]. According to Steudle [34] and others [74,87], the hydraulic conductivity of the plant depends on the nature and intensity of the forces driving the movement of water within the plant. Transpiration through stomata determines the shoot demand for water and serves as the driving force for water movement. Nonetheless, few studies have reported greater sensitivity (i.e., faster decline) of the leaf hydraulic conductance, as compared with stomatal conductance (g_s_), in response to stress [16,88]. This suggests the interdependence of transpiration rates, hydraulic conductance and water potential (i.e., dynamic regulation of hydraulic conductance can influence transpiration dynamics via changes in leaf water potential). This observation turns our attention to the soil-plant-atmosphere signal transduction pathway, which induces stomatal responses, particularly under stress (e.g., hydraulic, osmotic, ABA). Additionally, changes in leaf hydraulic conductance such as alternations in the hydraulic conductance of MCs (as mentioned above) are confounded with stomatal flux when g_s_ is measured using steady-state and diffusion porometers, making it difficult to pinpoint the specific contributions of internal tissues and possibly leading to inaccurate estimates of stomatal aperture [68].

Guard cells respond to many internal and external signals. However, while their electrochemical core mechanism of ion movement and accumulation has been well characterized [89,90,91,92], the facilitated diffusion membrane osmosis (i.e., AQP regulation) of guard cells has received less research attention. Guard cells are symplastically isolated, as plasmodesmata between neighboring epidermal cells are absent or rare [93,94]. Therefore, it is likely that the movement of water through guard cells, although passive, is regulated by membrane water permeability mechanisms. An additional unresolved question is whether the water that exits the guard cells is allocated to neighboring subsidiary cells, the apoplast or lost through transpiration.

During stomatal opening, the movement of water into the guard cell is substantial to the point of exocytic addition of membrane, permitting up to a 25% increase in cell volume [95]. This massive transport of water across the guard-cell membrane raises the question of whether and to what extent AQPs are involved in the process. However, the number of studies linking AQP activity and stomatal movement (in particular, opening) is surprisingly low. In sunflower (*Helianthus annuus*), the guard-cell aquaporin SunTIP7 was suggested to facilitate water exit, being associated with a decrease in guard cell volume. SunTIP7 increased the osmotic water permeability of *Xenopus* oocytes and its transcript levels increase systematically during daily stomatal closure. Transcript levels of an additional AQP in this study, SunTIP20, remained constant during the day, indicating that SunTIP genes are differentially regulated within the same cell [96]. In *Zea mays*, stomatal PIP subfamily transcript levels generally followed a diurnal pattern [97].

A more recent study showed a direct effect of AQP (PIP2;1) on guard-cell P_f_. In that study, phosphorylation of PIP2;1 by OST1 (a protein kinase involved in guard-cell ABA signaling) induced a two-fold increase in the P_f_ of guard-cell protoplasts, supporting the theory that ABA-triggered stomatal closure requires an increase in the permeability of these cells to water [98]. In a study involving the observation of stomata in a whole-plant context, GC-specific expression of NtAQP1 had no significant effect on g_s_ under normal conditions or when NaCl was added to the irrigation solution [64]. Nonetheless, Grondin et al. [98] reported differences between a PIP2;1 knockout and Wild Type (WT) stomata behavior following ABA treatment, but not under control conditions. In addition, it has been suggested that the guard-cell TIP subfamily may act as a sensor that responds to changes in the osmotic gradient and is involved in the dilution of the cytosol as the volume of the guard cells changes [99]. High concentrations of extracellular Ca^2+^ may also affect stomatal aperture by directly influencing water channels to retard aperture change [100].

## 6. Possible Water-Related, Post-Translational Regulation of AQPs

AQPs facilitate the movement of water through membranes and play an integral part in maintaining water-balance in the plant. Therefore, we would expect that AQP responses to dynamic environmental changes would be rapid and regulated (at least in part) in response to water-related parameters (i.e., RWC, water potential). AQP responses to membrane tension (as reflecting cell volume) can be found not only among plants [101,102], but also among yeasts [103], rabbits [104] and humans [105]. The prompt responses in plant water status and water-related regulation of AQP are expected to be regulated by post-translation modifications and not by transcription regulation.

Generally, phosphorylation is considered to be a main mechanism by which AQPs are activated [106]. In spinach (*Spinacia oleracea*), phosphorylation of PIP by a protein kinase associated with the PM is dependent on both apoplastic water potential and submicromolar concentrations of Ca^2+^, suggesting that AQP plays a role in the regulation of cell turgor [107]. The phosphorylation of AQP in a Ca^2+^-dependent manner was also observed in the context of temperature-dependent water transport in tulip (*Tulipa gesnerina*) that allowed the flower to open [108]. Drought-related [109] and ABA-related [110] dephosphorylation of PIP each seem to cause the closure of the water-transporting AQP gate.

X-rays of the spinach PM AQPs in its closed and open conformations indicate that during drought stress, these PIPs close in response to the dephosphorylation of two highly conserved serine residues. An additional mechanism reported to induce AQP closure is reduction in cytosolic pH (due to flooding in this case), PIPs response to the protonation of a fully conserved histidine facing the cytosol [106]. To the best of our knowledge, there have been no reports of any AQP response to apoplastic pH, yet fungal AQPs have been reported to play a role in regulating spore germination in acidic media (aquaporin activity increased with decreasing external pH), thanks to two histidine residues, positioned on two loops facing the outer side of the cell [111]. In addition to protons, divalent cations have also been shown to gate PIP [112,113].

Aquaporin re-localization (trafficking) from tonoplast to vesicular membranes as a result of mannitol-induced water imbalance [114] and salt treatment [115] has also been demonstrated. The formation of heterotetramers (AQP quaternary structures consist of tetramers) altering AQP activity was suggested as a post-translation modification that might alter water transport capacity [116,117,118,119], but the signal that might trigger such regulation requires further study.

Other water- and stress-related parameters may affect AQP function via additional mechanisms that are not yet understood. Such mechanisms may include: (1) changes in water flow intensity across the channel [102], with mechanical input perceived as the input of kinetic energy to the channel, which causes a conformational change of the protein, or the creation of tension at the constriction, in a manner analogous to Bernoulli’s principle for macroscopic pores (cohesion-tension model); (2) changes in cell turgor [59,101,120]; (3) concentration and molecular size of osmotic solutes [121] as described by the cohesion-tension model, with the size and concentration of solutes excluded from AQPs affecting whether they are open or closed; and (4) divalent cations and Ca^2+^ in particular [113], decreasing P_f_ of Arabidopsis cells in suspension.

For mechanical/physical parameters such as turgor, water flow across the channel and the size of osmotic solutes, the alteration of the protein conformation by a mechanical signal is the proposed mechanism triggering channel-gating [102,121,122]. Yet, these mechanisms are still unclear. For a summary of water-related post-translational regulation of AQPs, please see Table S1.

## 7. Conclusions

In this review, we have discussed several key control points along the xylem-to-stomata water pathway and suggested a possible role for AQP in the regulation of the flow along that route. Our composite model of water movement in the leaf includes parallel apoplastic, symplastic and transcellular water pathways through which water can be dynamically re-distributed, with that distribution regulated at least in part by AQP. AQP may provide a dynamic hydraulic adjustment to the fixed anatomical arrangement of the leaf, in response to the dynamic environment. We also propose a larger role for the mesophyll in regulating leaf hydraulics via the physical disconnection of the apoplast from the symplast: the hydraulic fuse theory. The plant’s ability to move water via different pathways in response to environmental signals may play a key role in the plant’s interactions with its environment. Together with stomatal regulation, this ability enables a wide range of plant hydraulic plasticity. An improved understanding of the role of AQPs in this mechanism could be useful for the development of crops with greater tolerance of abiotic stress.

## Supplementary Materials

Supplementary materials can be found at http://www.mdpi.com/1422-0067/17/7/1045/s1.
