# Supplementary material for: Role of Aquaporins in a Composite Model of Water Transport in the Leaf
Source: Int J Mol Sci. 2016 Jun 30;17(7):1045. doi: 10.3390/ijms17071045 (PMC4964421; doi:10.3390/ijms17071045)
Supplement: Supplementary file 1 [file ijms-17-01045-s001.pdf]

Supplementary Materials: Role of Aquaporins in a Composite Model of Water Transport in the Leaf

Adi Yaaran and Menachem Moshelion

Table S1. Possible water-related, post-translational regulation of AQPs.

	Trigger	Research Approach/Findings	Suggested Mechanism	Reference
Membrane	Changes in cell turgor	Hydraulic conductivity of *Zea mays* parenchyma cells increased as light intensity increased. Yet, when transportation reduce cells turgor (more than 0.2 MPa reduction) L_p was reduced despite light.	AQP-gating (light and turgor).	[59]
	Membrane tension/changes in cell turgor	Membrane trafficking inhibitors inhibited the rate at which the volume of intact *Vicia faba* guard cells decreased following 0–1.5 MPa osmotic treatments.	None.	[101]
	Membrane tension	Pulses of turgor pressure on individual young *Zea mays* root cortex cells decreased the hydraulic conductivity (L_p) of those cells. Cells with high L_p responded to $HgCl_2$.	Inhibition was in proportion with the absolute value of water flow within the channel, possibly due to a mechanical stimulus.	[102]
	Membrane surface tension/changes in cell turgor	Osmotic permeability (P_f) and activation energy for water transport (E_a) of TIP2;1 grapevine (*Vitis vinifera*) were dependent on internal turgor pressure.	TIP2;1 acts as a gate in an internal pressure-dependent manner.	[120]
Stresses	Drought stress/drop in cytoplasmic pH (anoxia due to flooding)	X-rays of spinach (*Spinacia oleracea*) SoPIP2;1 in its closed and open conformation; molecular dynamic simulations.	Dephosphorylation of two conserved serine residues; protonation of a conserved histidine residue.	[106]
	Long-term drought	Dephosphorylation of PIP2s in tobacco (*Nicotiana tabacum*) leaves exposed to long-term drought; $HgCl_2$ decreased the water permeability of the leaf tissues of well-irrigated leaves.	AQP-gating (closed conformation) by dephosphorylation.	[109]
	ABA	Phosphoproteome analysis following ABA treatment revealed decreased phosphorylation of four members of the *Arabidopsis thaliana* PM AQP family.	AQP-gating (closed conformation) by ABA-dependent dephosphorylation.	[110]
	Apoplastic water potential/turgor	Osmotic stress changes AQP phosphorylation in spinach (*Spinacia oleracea*), in a manner dependent upon submicromolar concentrations of Ca^{2+}. AQP transcript levels are high in vascular tissue.	Phosphorylation by PM-associated protein kinase is strictly dependent on submicromolar concentrations of Ca^{2+}.	[107]
	Osmotic stress	Mannitol-induced water imbalance resulted in increased AQP in tonoplast fractions of ice plants (*Mesembryanthemum crystallinum*), as well as a shift in AQP distribution in other membrane fractions. Redistribution of McTIP1;2 was limited by trafficking inhibitors.	AQP re-localization suggests a role for glycosylation and the involvement of a cAMP-dependent signal in the redistribution of McTIP1;2.	[114]
	Osmotic gating	Inhibition of AQP activity in isolated *Chara coralline* internodes increased with as the concentration and molecular size of solutes increased (reflection coefficients).	Cohesion/tension model of AQP-gating.	[121,122]
	Salinity	Exposure of *Arabidopsis thaliana* roots to salt induces the redistribution of AQP to intracellular spherical structures tentatively identified as intravacuolar invaginations.	Subcellular re-localization of PIPs and TIPs.	[115]

Table S1. *Cont.*

	Trigger	Research Approach/Findings	Suggested Mechanism	Reference
Ion signals	Apoplast pH	$HgCl_2$ prevented acidity induces swelling and floating of *Rhizopus delemar*s spores.	pH-dependent AQP gating via histidine residues on the outer surface of the cel.l	[111]
	Ca^{2+} and H^+	Adding Ca^{2+}caused a 4-fold reduction in the hydraulic conductivity of *Arabidopsis thaliana* cells in suspension. The ability of purified plasma-membrane vesicles to transport water was reversibly blocked by $Ca^{2+}$and H^+.	Ca^{2+} and H^+ are involved in AQP-gating.	[113]
	Divalent cations and protons	Cd^{2+} and Mn^{2+} were also identified as potent inhibitors of *Pichia pastoris* AtPIP2;1S, showing dose-dependent effects on proteoliposomes containing AtPIP2;1.	Specific residues are involved in binding divalent cations and/or stabilizing open- or closed-pore conformations.	[112]
Temperature	Temperature change (5 °C to 20 °C)	Incorporation of ^{32}P into AQP in microsomal membranes of tulip (*Tulipa gesnerina*) petals was temperature- and time-dependent.	Phosphorylation of AQP by PM associated protein kinase is dependent on the concentration of Ca^{2+}.	[108]
Unknown	?	Co-expression and physical interaction of nonfunctional and functional ZmPIPs resulted in changes in the permeability of *Xenopus* oocytes to water, based on the amount of injected complementary RNA.	Formation of alternative heterotetramers alters AQP activity.	[116]
	?	Artificial heterotetramers with a defined proportion of NtAQP1 to tPIP2;1 tetramer affected the transport of water in yeast.	Formation of alternative heterotetramers alters AQP activity.	[119]
	?	MpPIP1;1 (which exhibits no water-channel activity) facilitated the activity of MpPIP2;1 in a phosphorylation-dependent manner.	Cooperative regulation of MpPIP2;1 was regulated by phosphorylation of MpPIP1;1.	[117]
